# Comparative Efficacy and Safety of Deferoxamine, Deferiprone and Deferasirox on Severe Thalassemia: A Meta-Analysis of 16 Randomized Controlled Trials

**DOI:** 10.1371/journal.pone.0082662

**Published:** 2013-12-23

**Authors:** Sujian Xia, Weidong Zhang, Liting Huang, Hong Jiang

**Affiliations:** 1 Division of Medical Statistics, School of Medicine, University of Jinan, Guangzhou City, Guangdong, China; 2 Novartis Pharmaceuticals Oncology, Beijing, China; 3 Zhuhai People’s Hospital, Zhuhai City, Guangdong, China; University of Illinois at Chicago, United States of America

## Abstract

**Objective:**

A meta-analysis was conducted to investigate the efficacy and safety of three main iron chelators, namely, deferoxamine (DFO), deferiprone (DFP) and deferasirox (DFX) for thalassemia major (TM) patients.

**Methods:**

Randomized controlled trials comparing mono-therapy DFO, DFP, DFX and combined DFP with DFO therapy in TM patients from January 1990 to December 2012 were searched and selected. Two independent authors assessed data from extracted randomized trials for efficacy and safety in the measurements of serum ferritin (SF), live iron concentration (LIC), myocardial iron content (MIC), left ventricular ejection fraction (LVEF) and adverse events (AEs).

**Results:**

Sixteen studies were selected. In the comparison of DFP versus DFO treatment groups, a significant difference was revealed on MIC and LVEF (*P*=0.01 and *P*=0.007, respectively) but not on SF or LIC level (*P*=0.65 and *P*=0.37, respectively). In comparing combined therapy (DFP plus DFO) versus DFO, a significant difference was shown on MIC and LVEF measurements (*P*<0.00001 and *P*=0.003, respectively), but not on SF or LIC levels (*P*=0.93 and *P*=0.62, respectively). Moreover, the combined DFP with DFO treatment had significantly higher risk than DFO treatment (RR 1.46 with 95%CI 1.04 to 2.04). When comparing DFX with DFO, a significant difference was shown on the SF level (*P*=0.003), and there was no difference between DFX and DFO in safety evaluation (RR 1.53 with 95%CI 0.31 to 7.49).

**Conclusion:**

Findings indicated that the most effective and safe iron chelators remains to be proven, and further large-scale, long-term studies are needed.

## Introduction

Thalassemia is a severe genetic blood disorder caused by a mutation in the globin gene leading to the excessive destruction of red blood cells [1]. It has been estimated that over 42 000 newborns are affected by Beta-thalassemia every year worldwide. Without any aid, such as blood transfusion, Beta-thalassemia major (TM) causes death amongst infected children before the age of 3 years old [2]. Regular blood transfusions can prevent death and decrease mortality. However, excessive iron accumulated from transfused red blood cells can lead to organ failure [3,4]. Therefore, iron chelation treatment, which can reduce iron store in the body and improve the long-term survival rate of patients with TM, is considered necessary adjuvant therapy. 

 Currently, the main iron chelators available for clinical use are desferrioxamine (DFO), deferiprone (DFP) and deferasirox (DFX). DFO, which requires routine subcutaneous or intravenous injection on 5 to 7 days per week, is considered a standard treatment for iron overload during the past four decades. However, the treatment with DFO for 8 to 12 hours per day results in poor compliance and some negative impacts such as skin rash, hematological toxicity and heart failure [5,6].

 DFP, expected to be a great improver over DFO, has been licensed to treat patients who were inadequately treated with DFO in Asia and Europe since 1990s. But the use of DFP has not been approved in North America [7,8]. Although DFP has good compliance, some serious side effects such as gastrointestinal disturbances, arthropathy, neutropenia and agranulocytosis were reported. However, the combination of DFP and DFO, regarded as “shuttle hypothesis”, was hoped to have a synergistic effect on iron removal and patient compliance [9]. 

 DFX, a once-daily oral iron chelator, was introduced as first line therapy for patients over 2 years of age with chronic iron overload due to blood transfusions in 2005. Although DFX has some mild adverse events, some studies indicated that it has a positive effect on lowering liver iron and producing high patient compliance [10,11].

 A large amount of studies have been conducted to assess the efficacy and safety of aforementioned three iron chelators. However, results are presented from independent research with different study designs and diverse formulations of drugs for the treatment of iron overload. Up to now, it remains unclear what the evidence-based the standardized chelation protocol is. Therefore, it is necessary to conduct a meta-analysis of published studies to assess the efficacy and safety of iron chelators in TM. 

## Methods

### Study Sources and Searches

The literature search was conducted in Medline, PubMed, Embase, ISI website of knowledge and The CENTRAL of Cochrane Library to identify relevant published English articles from January 1990 to December 2012. The search key words and subject terms were used included “deferoxamine” “deferiprone” “deferasirox” “iron chelators” and “thalasseamia major”. Relevant articles in reference lists of published articles were also searched. 

### Study selection and data extraction

All studies that were identified by the literature searches were reviewed and selected according to the following prior criteria: (i) patients with thalassemia major regardless of age and sex; (ii) randomized controlled trials (RCTs) with at least two groups comparing with DFO, DFP, DFX or the combination of DFP and DFO; and (iii) outcomes of iron storage or adverse effects in patients. Authors of selected studies were contacted for further information when necessary.

 Data was extracted by two independent reviewers. The extracted information included: (i) the first author, published year, study type and study duration; (ii) the number and characteristics of subjects; (iii) drug regimens, doses and treatment duration; and (iv) outcomes. The two reviewers reached agreement on selected articles and extracted information and if disagreed, a third reviewer was invited to resolve the differences.

### Evaluation of methodological quality

To assess the validity of RCTs studies, the Risk of Bias Tool [12]evaluation was conducted following the recommendations from the Cochrane collaboration. The Tool includes six domains as follows: selection bias (random sequence generation and allocation concealment), performance bias (blinding of participants and personnel), detection bias (blinding of outcome assessment), attrition bias (incomplete outcome data), reporting bias (selective reporting) and other bias. Each of the six domains was assessed to reach a judgment categorized as “low risk”, “high risk”, or “unclear”. “Unclear” was used if there was insufficient information to make an informed judgment.

### Statistical analysis

Meta-analysis was performed via RevMan 5. The efficacy of three chelators were evaluated via two main measurements (serum ferritin [SF] and live iron concentration [LIC]) and two important cardiac measurements (myocardial iron concentration [MIC] and left ventricular ejection fraction [LVEF]). For continuous outcomes with the same measurement scale, mean difference (MD) or weighted mean difference (WMD) were computed with 95% confidence intervals (CIs). However, for continuous outcomes with different measurement scales, standardized mean difference (SMD) was calculated. The safety of the three chelators were measured using the number of adverse events (AEs). The dichotomous outcomes were presented as a risk ratio (RR) with 95% CIs. 

 The heterogeneity between trails was tested by using the Cochrane’s Q test and *I* ^2^ statistic. A *I* ^2^ value of 40% or lower was considered a low heterogeneity and a value up to 60% was represented a moderate heterogeneity. When the heterogeneity was significant (*P*≤0.10) but the studies’ characteristics were similar enough, the random effect model was used for combining data. When the *I* ^2^ value was over 75%, it suggested a substantial heterogeneity and the combination analysis would be performed with caution if there was no obvious clinical or methodological difference among trails. When there was no apparent heterogeneity (*P*>0.10), a fixed effect model was applied [12]. Outcomes were summarized and expressed using a forest plot. Descriptive analysis was used for individual result which is not available for meta-analysis.

## Results

### Search results

**Figure 1:**
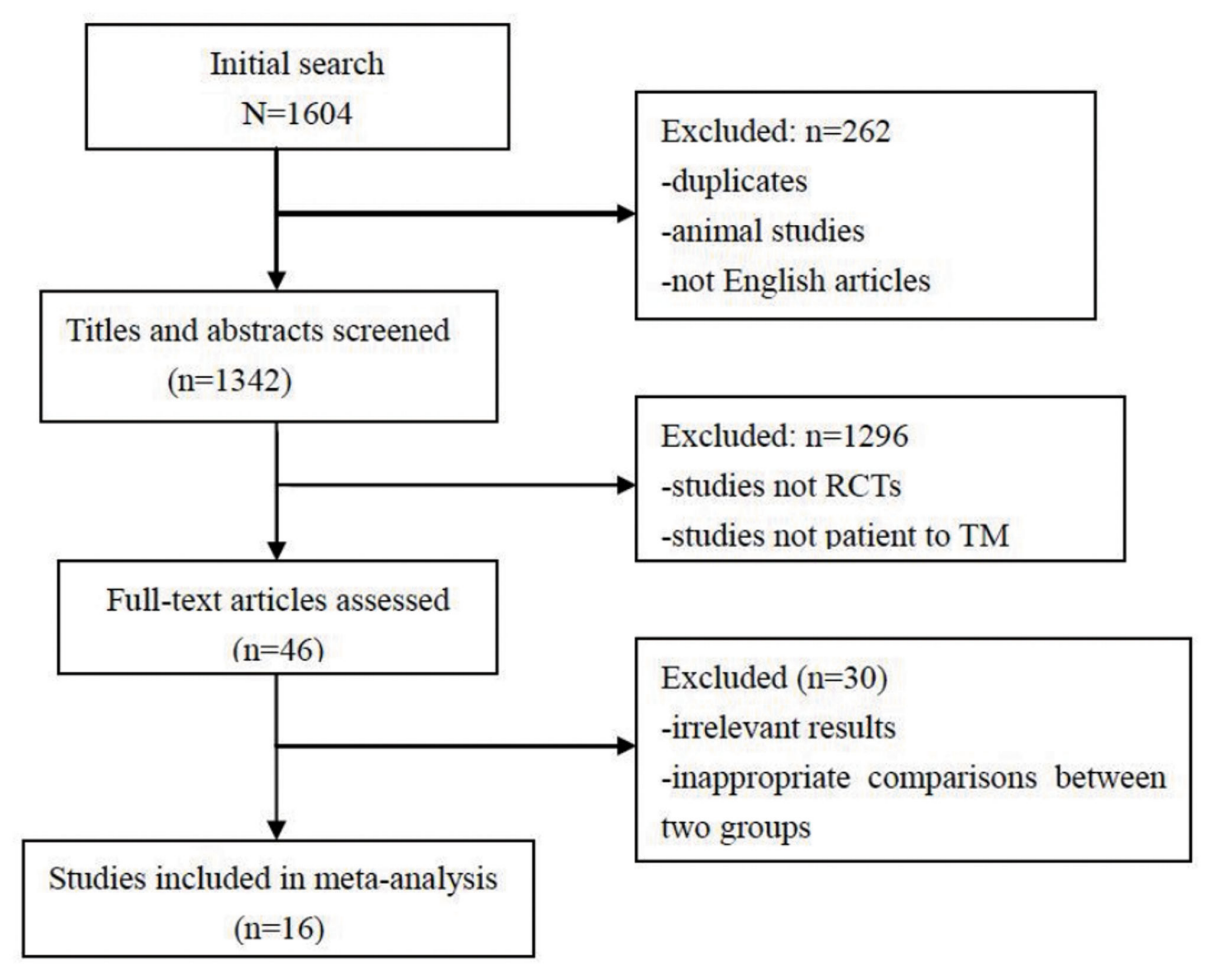
Flow diagram of study selection process.

Sixteen RCT studies were included in this meta-analysis with a total of 1,194 patients. The flow diagram of the study search process is presented in Figure 1. Out of the 24 included RCTs, 9 RCTs compared DFP versus DFO [13-21], 6 DFX versus DFO [22,23], 7 combined DFP with DFO versus DFO [13,15,17,24-27]and 2 sequential DFP with DFO versus DFO [28,29]. (Table 1) 

**Table 1:** Types of interventions, treatment duration and number of participants of included studies.

Study [ref]	Exp	Exp N. pts	Ctrl	Ctrl N.pts	Duration(m,)
Gomber *et al* (2004)	DFP	11	DFO	7	12
Pennell *et al* (2006)	DFP	27	DFO	32	12
El-beshlawy *et al* (2008)	DFP	18	DFO	20	12
Galia *et al* (2003)	DFP	37	DFO	35	12
Maggio *et al* (2002)	DFP	71	DFO	73	12
Smith *et al* (2011)[19]	DFP	27	DFO	29	12
Aydinok *et al* (2007)	DFP	12	DFO	12	12
Ha *et al* (2006)	DFP	6	DFO	7	18
Peng *et al* (2003)	DFP	11	DFO	10	36
Cappellini *et al* (2006)	DFX(5mg)	15	DFO	14	12
Piga *et al* (2006)	DFX(10mg)	24	DFO	23	1.5
Cappellini *et al* (2006)	DFX(10mg)	78	DFO	79	12
Piga *et al* (2006)	DFX(20mg)	24	DFO	23	1.5
Cappellini *et al* (2006)	DFX(20mg)	84	DFO	91	12
Cappellini *et al* (2006)	DFX(30mg)	119	DFO	106	12
Ha *et al* (2006)	DFP-DFO	17	DFO	14	18
Mourad *et al* (2002)	DFP-DFO	11	DFO	14	12
Tanner *et al* (2007)	DFP-DFO	28	DFO-Placebo	30	12
Aydinok *et al* (2007)	DFP-DFO	8	DFO	12	12
Gomber *et al* (2004)	DFP-DFO	10	DFO	7	12
El-beshlawy *et al* (2008)	DFP-DFO	18	DFO	20	12
Galanello *et al* (2006)	DFP-DFO	29	DFO	30	12
Maggio *et al* (2009)	Sequential DFP-DFO	105	DFP	108	60
Pantalone *et al* (2011)	Sequential DFP-DFO	36	DFP	27	60

### Risk of bias

Of 16 RCTs, the random sequence generation was clearly described in 9 (56.3%) RCTs. Concealed allocation was used in 4 (25.0%) studies. Three (18.8%) studies were double-blinded for participants and personnel as well as outcome assessment. Only one study [24] presented the completed outcome data 3 (18.8%) and 8 (50.0%) studies had a low risk of bias on selective outcome reporting and other sources of bias, respectively (Table 2). 

**Table 2:** Risk of bias chart for each study.

Study	Sequence generation	Allocation Concealment	Blinding of participants and personnel	Blinding of outcome assessment	Incomplete outcome data	Selective reporting	Other bias
Aydinok et al (2007)	L	H	L	N	H	H	L
Cappellini et al (2006)	L	N	H	H	H	H	L
El-beshlawy et al (2008)	N	N	N	N	H	H	H
Galanello et al (2006)	L	L	N	N	H	H	N
Galia et al (2003)	L	L	N	N	N	H	N
Gomber et al (2004)	N	N	N	N	H	H	L
Ha et al (2006)	L	H	H	H	H	H	L
Maggio et al (2002)	L	L	N	N	H	L	L
Maggio et al (2009)	L	L	L	L	H	N	L
Mourad et al (2003)	N	N	N	N	L	H	N
Pantalone et al (2011)	H	N	L	L	N	N	N
Peng et al (2003)	H	H	N	H	N	N	N
Pennell et al (2006)	N	N	N	N	H	H	L
Piga et al (2006)	L	N	N	H	H	H	H
Simth et al (2011)	L	N	N	L	N	L	N
Tanner et al (2007)	N	N	L	N	N	L	H

Notes: L, low risk; N, unclear; H, high risk.

### Meta-analysis of outcomes

All outcome variables were presented as MD or SMD with 95% confidence intervals by meta-analysis, while single study and studies did not provide in means and SDs data were assessed by descriptive analysis. If the required measures were not reported in the original paper, the original data were requested the authors and relevant measures were derived using SPSS version 13.0.

### Difference in Serum ferritin (SF) between the baseline and the end of the intervention

Eleven studies presented the mean and standard derivation (SD) of the differences in SF level between the baseline and the end of the intervention. 

**Figure 2:**
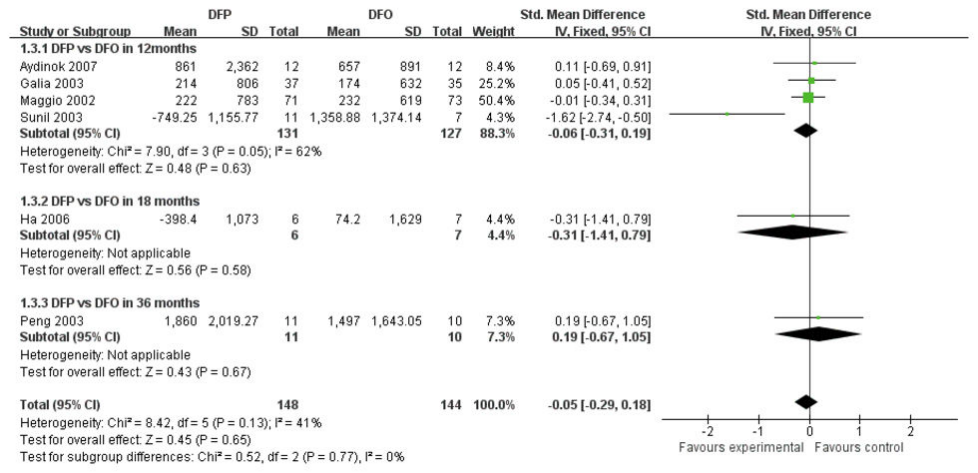
Forrest plot for the decrease of SF between DFP group and DFO group.

Six trials [13,16-18,20,21] with DFP and DFO comparison were divided into three subgroups according to their treatment durations. The combined analysis of three subgroups indicated that there was no significant difference between DFP group and DFO group (SMD -0.05, 95%CI -0.29 to 0.18, *P*=0.65). (Figure 2). 

**Figure 3:**
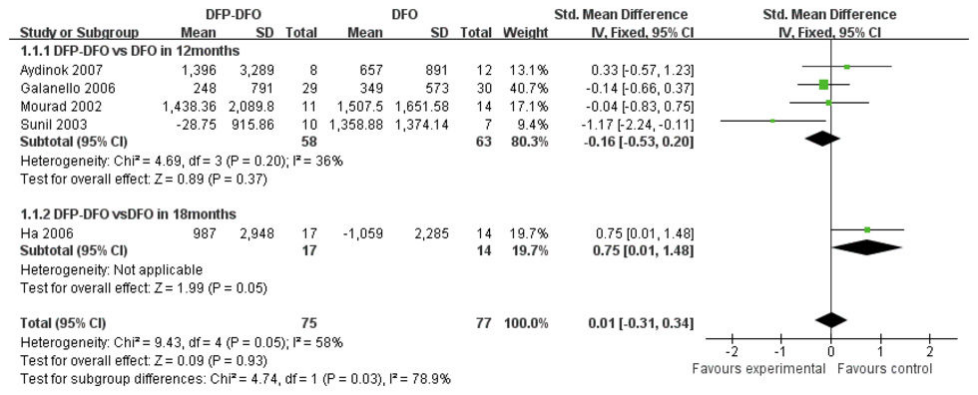
Forrest plot for the decrease of SF between DFP plus DFO group and DFO group.

In five studies, DFP plus DFO was compared with DFO. Four of them were included in a subgroup analysis at 12-month treatment duration, while one study was assessed at 18 months. The heterogeneity was at the moderate level in the four studies group (*P*=0.05, *I* ^2^=58%). The overall effect of two subgroups indicated that there was no statistically significant difference between the two iron chelation regimens in SF level (SMD 0.01, 95%CI -0.31to 0.34, *P*=0.93). Specifically, the standard mean difference of subgroups at 12 months was -0.16 (95%CI -0.53 to 0.20, *P*=0.20), which indicating a non-significant difference between intervention and control groups. However, Ha [17] reported that the combination therapy significantly reduced the SF level compared with DFO treatment group (SMD 0.75, 95%CI 0.01 to1.48, p=0.05). (Figure 3)

**Figure 4:**
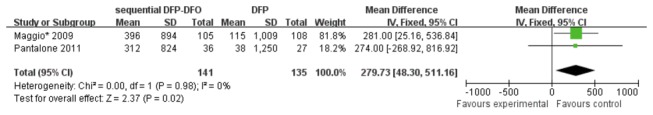
Forrest plot for the decrease of SF between sequential DFP with DFO group and DFP group.

Combined analysis of two trials of comparing sequential DFP plus DFO versus DFP showed no heterogeneity (*P*=0.98, *I* ^2^=0%). The reduction of SF in sequential DFP plus DFO group was significant greater than DFP group (MD 279.73, 95%CI: 511.16 to 48.30, *P*=0.02). (Figure 4)

**Figure 5:**
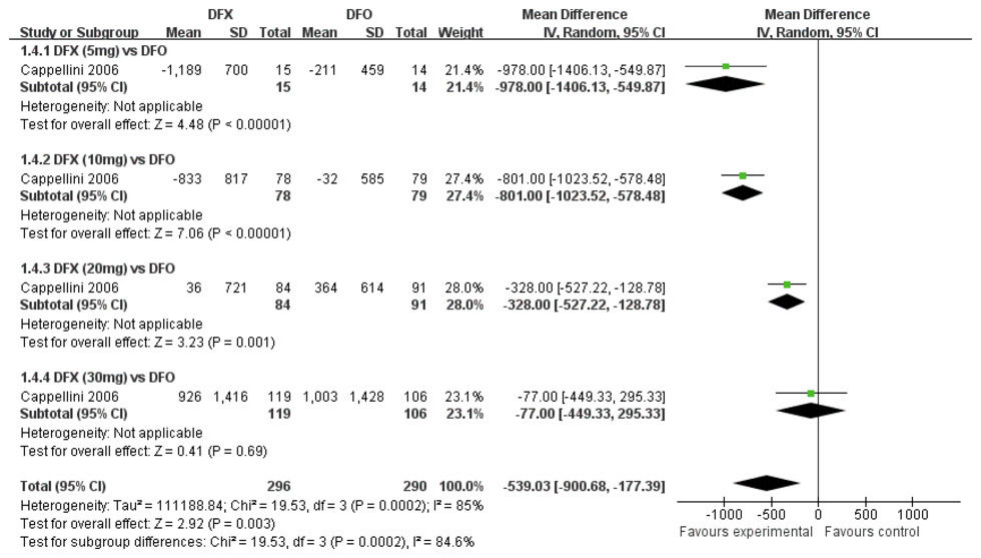
Forrest plot for the reduction of SF between DFX group and DFO group.

One study [23] compared DFX and DFO. Subgroup analysis was performed based on different doses of DFX. There was a high heterogeneity among the four subgroups significantly (*P*< 0.01, *I* ^2^=84.6%). To summarize the effects, the change of SF in DFX treatment group was more significant than that in DFO treatment group (MD 539.03, 95%CI: 177.39 to 900.68, *P*=0.003). Specifically, the reduction of SF level was not observed when patients received 5mg/kg or 10 mg/kg DFX until receiving 20mg/kg and 30mg/kg DFX. (Figure 5)

### Difference in liver iron concentration (LIC)between the baseline and the end of the intervention

**Figure 6:**
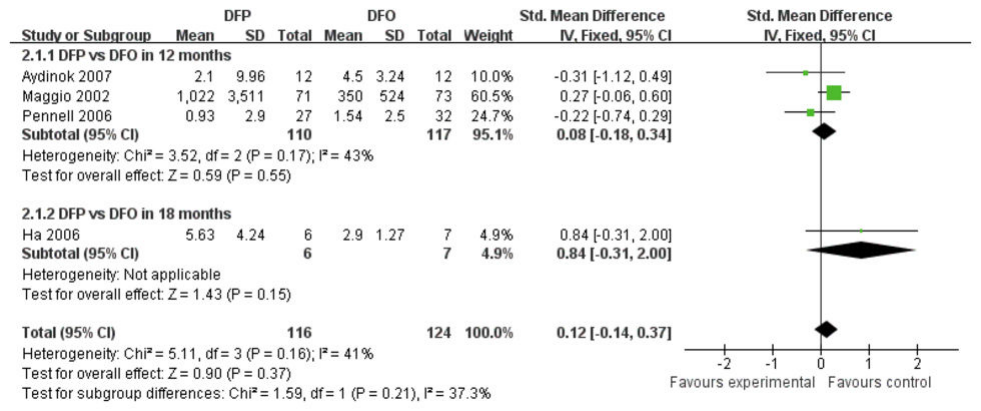
Forrest plot for the decrease of LIC between DFP group and DFO group.

Eight studies with 936 patients reported the outcome of LIC. According to different durations, four studies with DFP and DFO comparison were divided into two subgroups: three studies with a 12-month duration and one with an 18-month duration. There was a low heterogeneity among these two groups (*P*=0.16, *I* ^2^=41%). The pooling analysis of two subgroups indicated that there was no statistically significant difference in LIC level between DFP treatment group and DFO treatment group (SMD 0.12, 95%CI: -0.14 to 0.37, *P*= 0.37). (Figure 6)

**Figure 7:**
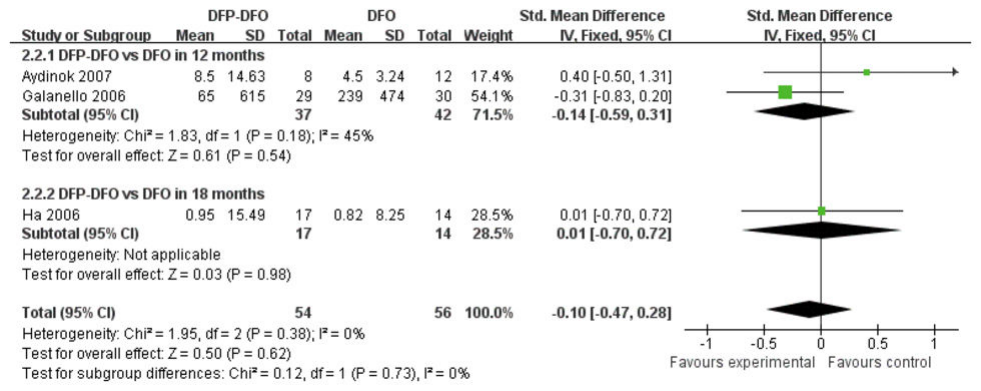
Forrest plot of the difference on the decrease of LIC between combined DFP with DFO group and DFO group.

Three trials compared LIC levels between DFP plus DFO versus DFO. It showed that the difference in LIC levels between the two treatment groups was not statistically significant (SMD -0.10, 95%CI: -0.47 to 0.28, *P*=0.62). (Figure 7)

**Figure 8:**
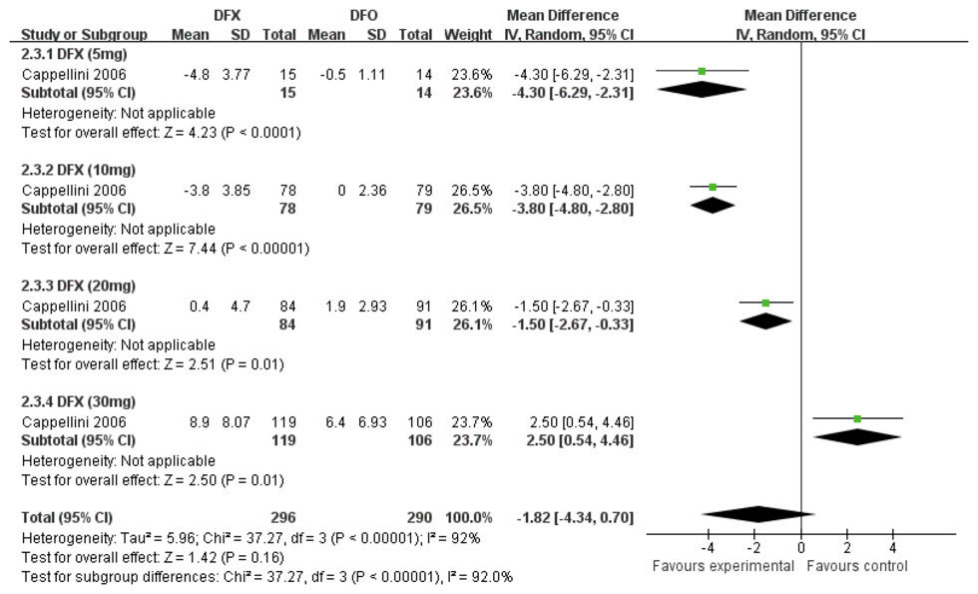
Forrest plot of the difference on the decrease of LIC between DFX group and DFO group.

One study [23] compared the LIC levels between DFX and DFO by four doses. Meta analysis in Figure 8 showed that there was a substantial heterogeneity among four doses groups (*P*<0.00001, *I* ^2^=92%). The reduction in LIC did not showed in DFX with 5mg or 10mg treatment groups but showed in DFX with 20 mg and 30mg treatment groups. Moreover, the reduction in patients with 30mg/kg DFX was a significantly difference from that in DFO treatment group (MD 2.50, 95% CI: 0.54 to 4.62, *P*=0.01). One study [22] showed that the reduction in LIC level was similar in both DFX 20mg/kg and DFO groups (-2.1 and -2.0 mg Fe/g dw, respectively). (Figure 8)

### Outcomes of myocardial iron concentration (assessed by MRI T2*)

Five studies reported myocardial iron concentration via MRI T2* test.

**Figure 9:**
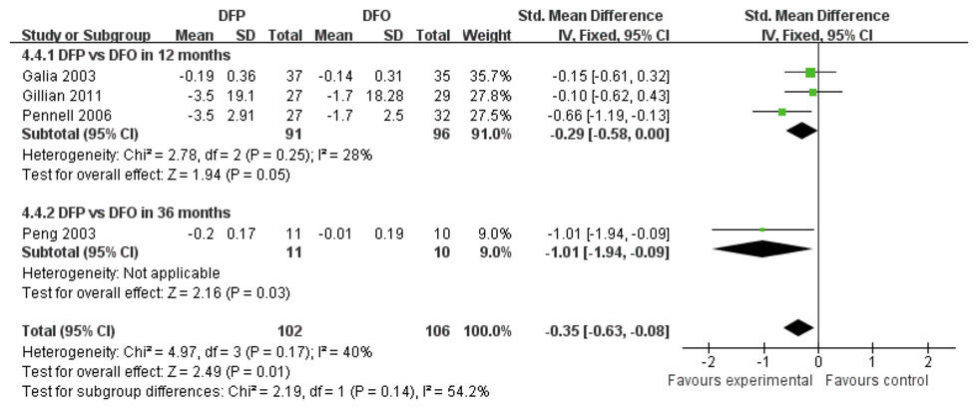
Forrest plot of the difference on MRI for myocardial iron concentration between DFP group and DFO group.

The comparison of DFP and DFO was divided into two subgroups based on different treatment durations (Figure 9). There was a statistically significant difference in summary effect of myocardial iron concentration between DFP and DFO treatment groups (SMD -0.35, 95% CI: -0.63 to -0.08, *P*=0.01).

In comparison of combined DFP/DFO therapy versus DFO only therapy, Tanner [25] showed that the combination therapy in MIC was more effective than mono-therapy significantly (SMD 2.68, 95% CI: 1.96 to 3.40, *P*<0.00001). 

### Outcomes of left ventricular ejection fraction (LVEF)

**Figure 10:**
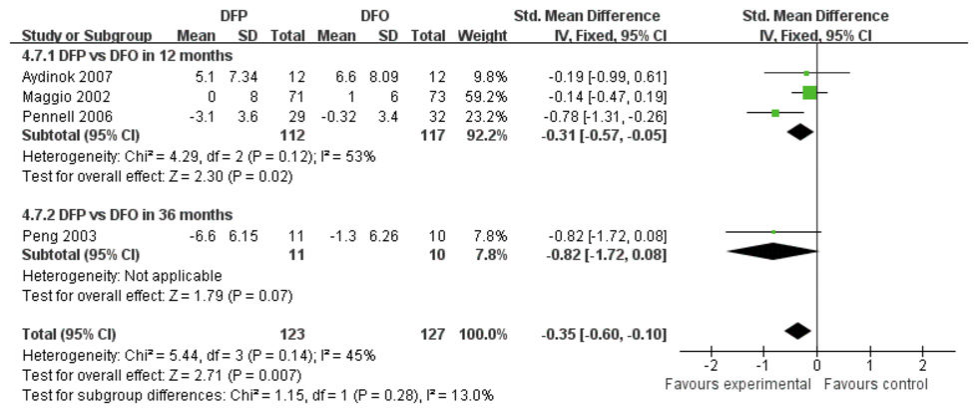
Forest plot of outcomes of LVEF as difference of means and standard deviations from before and after the study between DFP and DFO.

**Figure 11:**
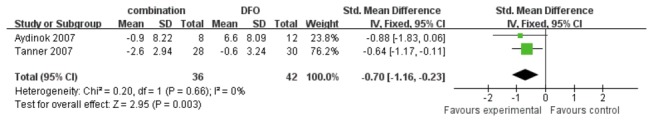
Forest plot analysis of outcomes of LVEF as difference of means and standard deviations from before and after the study between combination therapy and DFO.

Five trials reported outcomes of LVEF (Figures 10-11). A low level heterogeneity was observed in the studies comparing DFP and DFO (*P*=0.14, *I* ^2^=45%). Figure 10 showed that the reduction of LVEF in DFP treatment groups was a significantly different from that in DFO treatment group (SMD -0.35, 95%CI: -0.60 to -0.10, *P*=0.007). Figure 11 showed that there was no heterogeneity between combination treatment group and DFO treatment group (*P*=0.66, *I* ^2^=0%). A statistically significant difference was observed between combination and DFO treatment groups (SMD -0.70, 95% CI: -1.16 to -0.23, *P*=0.003). 

### Safety evaluation

**Figure 12:**
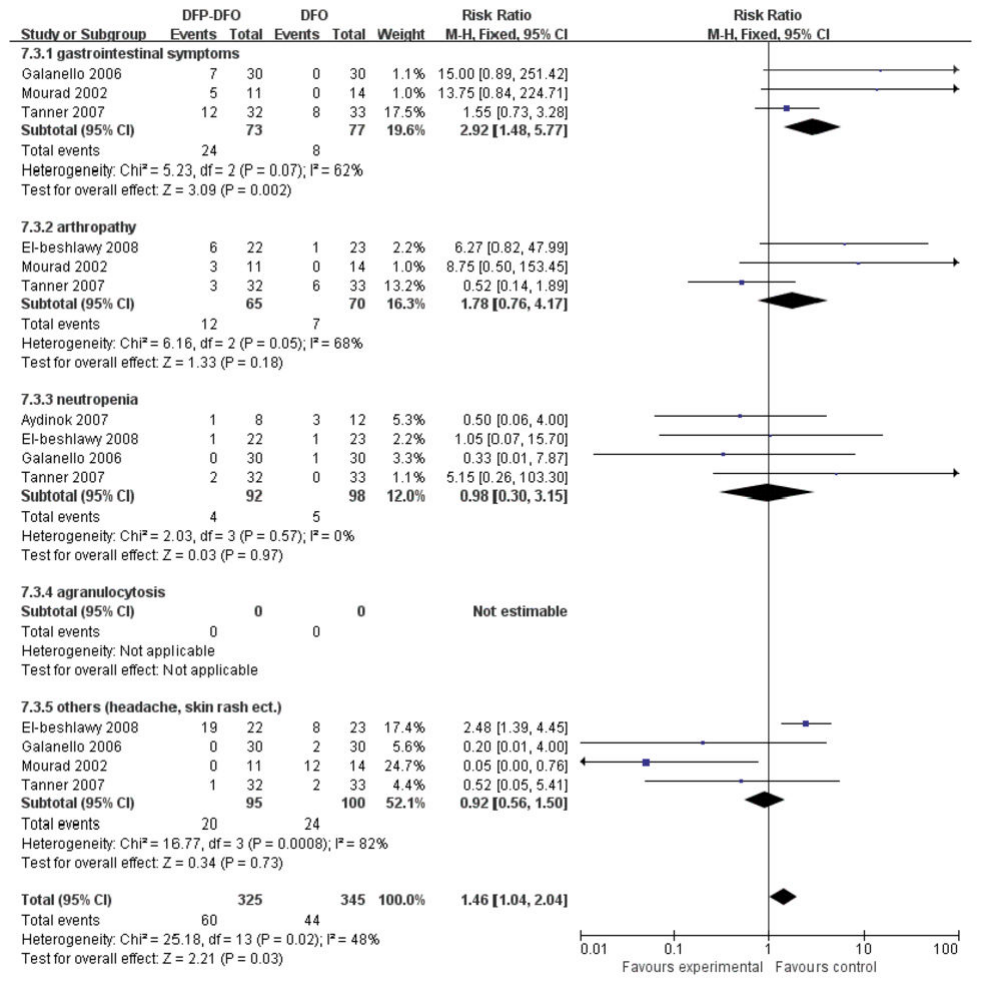
AEs of the comparison of DFP plus DFO versus DFO treatment group.

**Figure 13:**
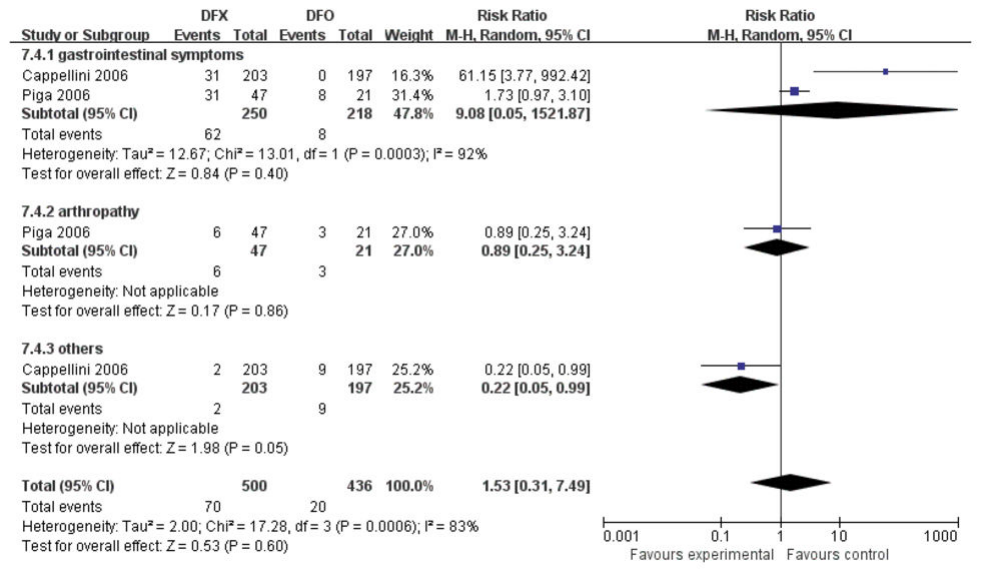
AEs of the comparison of DFX versus DFO.

Fourteen out of 20 studies reported adverse events (AEs) with different iron regimens. Two paired comparisons of treatment groups (ie, combination of DFP and DFO versus DFO and DFX versus DFO) were considered. The main AEs mentioned in such studies included mild-to-moderate events like gastrointestinal symptoms and arthropathy, and severe events such as neutropenia and agranulocytosis. Study groups were allocated according to difference comparison trials and subgroups were classified based on different AEs as shown in Figures 12-13.

Fourteen trails were involved in the analysis of AEs with the comparison therapy and DFO (Figure 12). A statistically low heterogeneity among 14 studies was observed (*P*=0.02, *I* ^2^=48%), Indicating that the risk ratio between DFP plus DFO treatment group and DFO treatment group was apparently high (RR=1.46, 95% CI: 1.04 to 2.04, *P*=0.03). 

Two studies [22,23] comparing DFX and DFO were identified and assessed (Figure 13). A significantly high heterogeneity was presented among four trials (*P*=0.0006, *I* ^2^=83%). The risk ratio between DFX and DFO treatment groups was not significantly high (RR=1.53, 95% CI: 0.31 to 7.49, *P*=0.60). 

## Discussion

For transfusion-dependent TM patients, it is important to choose an appropriate iron chelator to reduce iron burden in the body in order to prolong life and improve the quality of life. Although DFO was considered as “gold standard” for the last three decades, clinical experience demonstrated that parenteral DFO treatment was insufficient to reduce cardiac iron burden and had low compliance in patients [30]. In the present study, a meta analysis was conducted using a total of 16 RCTs to evaluate the efficacy and safety of iron chelators. 

### Efficacy

To evaluate the efficacy of iron chelators, relative treatment effects of each therapy were assessed by comparing differences in means and standard deviations. SF and LIC measurements have been considered as principle methods for measuring total body iron stores [31]. This meta-analysis demonstrated that either DFP only or combined with DFO did not have a significantly different effect on SF level compared with DFO-only treatment. It suggests that the combination therapy (DFP and DFO) and mono-therapy DFP are as effective as DFO treatment on the SF level. However, Gomber [13] showed that DFO was significantly effective to reduce iron overload whereas SF level still increased on DFP only or combined therapies. Such a difference could be attributable to small sample sizes. Two studies [28,29] with no heterogeneity indicated that the sequential DFP plus DFO treatment was more effective in reducing SF level than DFP-only treatment. Although no difference was found between DFP and DFO, it is unlikely that the sequential treatment is more effective than DFO treatment as no RCTs with such a comparison could be identified in the study search for this meta-analysis. Four trials [23] compared DFX treatment groups to DFO treatment groups with a high level heterogeneity. It may be due to different doses in four intervention groups (5, 10, 20 and 30mg/kg of DFX , respectively). The SF level in 5 and 10mg/kg groups slightly changed with 20mg/kg and significantly decreased in 30mg/kg. It suggests that effectiveness of DFX on reducing the SF level may require appropriate dose adjustment. However, this was observed only in one study. Additional studies and further evidences were needed to confirm such an observation. In terms of the LIC measurement, 6 studies [14,17,18,20,23,27] showed that mono-therapies DFP and DFO and associated DFP plus DFO therapy decreased LIC significantly. However, there were no significant differences among these three types of treatments. However, a systemic review [32] showed that DFO was more effective in reducing LIC level than DFP. Additional quality studies are needed to clarity this issue. Cappenllin [23] demonstrated that a dosage of 20 mg/kg DFX nearly unchanged LIC but a dosage of 30 mg/kg decreased LIC significantly. This suggests that a DFX dosage of 30my/kg is as effective as DFO in changing the LIC level as proven by Vanorden [33].

Nowadays, MRI T2* becomes an important standard for measuring cardiac iron due to its noninvasive nature compared with biopsy, and LVEF is another important parameter that is correlated with cardiac iron [1]. Seven RCTs [14,16,18-21,25] noted that treatments with DFP only or combined with DFO were more effective than DFO only on both MRI T2* and LVEF. It indicated that DFP mono-therapy and combination therapy are more effective on the improvement of cardiac function than DFO therapy. However, Mamtani and Kulkarni [34] showed there was no significant difference among DFO, DFP mono-therapy and combination therapy. It may be due to the difference in studies selected as in this paper only RCTs was included. In addition, heterogeneity may affect the results since Mantani and Kulkarni showed a statistically significant heterogeneity between DFP and DFO treatment groups while a non-significant heterogeneity (*I* ^2^=40%) was observed in our meta analysis. No data in selected studies showed the relationship between DFX and cardiac function. Although several non-RCT studies [35-37] suggested that DFX was effective to remove cardiac iron in patients. RCTs examining the effects of DFX on cardiac MRI are warranted to verify such a finding. 

### Safety

In the present study, a total of 7 studies [15,20,22-25,27] reported AEs that were meta-analyzed as safety measures. In comparing DFP plus DFO and DFO only treatments, patients with DFP plus DFP were likely to have gastrointestinal disturbances, whereas patients with DFO only were likely to have skin rash or other mild AEs. The causes of these AEs could be attributable to different modes of administration. DFP is an oral drug while DFO is taken via subcutaneous or intravenous injection. However, the incidence of arthropathy in combined therapy was higher than DFO monotherapy. Moreover, no patients in DFO group developed from neutropenia to agranulocytosis. However, two out of four cases of neutropenia developed into agranulocytosis in combined therapy [20,25]. These observations indicated that DFO could be safer than DFP. 

In the comparison of DFX and DFO, although two studies had a high heterogeneity, no significant difference in the occurance of AEs was seen between these two treatments. In addition, neither of two studies reported any cases with neutropenia and agranulocytosis. However, long-term parenteral infusions in DFO leads to poor compliance [31]. DFX, an orally active chelator, seems to have a reasonable safety compared with DFO. 

### Limitation

This meta-analysis study has several limitations. Firstly, although 16 articles were extracted, they presented different comparisons trials. The study samples of each comparison trials were not sufficient to assess as the funnel plot so that the publication bias of involved studies is unclear. Moreover, there is a risk bias that separate studies may have used the same sample source without being clearly identified [38]. Secondly, out of 16 studies, 7 studies did not report the random sequence generation, 12 studies did not presented the allocation concealment, and only 4 studies were double-blind. Thus, some studies had a high risk for bias. Thirdly, some results were based on very few studies and will need to be further proved by more related studies. Finally, it is unavoidable that different characteristics of patients, such as age and physical conditions, may lead to different results. 

In conclusion, the results of this meta-analysis from 16 RCTs suggest that treatment with DFP as mono-therapy or in combination with DFO improve cardiac function effectively. However, they may cause some severe adverse events. DFX is likely to have a safety profile as a once-daily oral drug; however, its long term effect for TM patients, especially on the cardiac function, is still unclear. Overall, the most appropriate chelation regimen remains to be proved and well designed and long-term RCTs are needed.

## Supporting Information

Checklist S1 **Checklist of meta-analysis of the Comparative efficacy and safety of deferoxamine, deferiprone and deferasirox on severe thalassemia.**
(DOC)
